# Prevalence survey on lungworm (*Angiostrongylus vasorum*, *Crenosoma vulpis*, *Eucoleus aerophilus*) infections of wild red foxes (*Vulpes vulpes*) in central Germany

**DOI:** 10.1186/s13071-018-2672-4

**Published:** 2018-02-06

**Authors:** Kathrin Schug, Friederike Krämer, Roland Schaper, Jörg Hirzmann, Klaus Failing, Carlos Hermosilla, Anja Taubert

**Affiliations:** 1 Institute of Parasitology, Justus Liebig University Giessen, Giessen, Germany; 2 Institute of Parasitology, Faculty of Veterinary Medicine, Leipzig University, Leipzig, Germany; 3 Bayer Animal Health GmbH, Global Marketing CAP, MON/6210, 51373 Leverkusen, Germany; 4 Unit for Biomathematics and Data Processing, Justus Liebig University Giessen, Giessen, Germany

**Keywords:** Fox, Dissection, Epidemiology, Hesse, Rhineland-palatinate, Thuringia, Germany, PCR

## Abstract

**Background:**

*Angiostrongylus vasorum*, *Crenosoma vulpis* and *Eucoleus aerophilus* are a source of increasing concern, potentially causing significant pulmonary and severe cardiac/systemic diseases in domestic dogs and wild canids, especially red foxes (*Vulpes vulpes*). To investigate the prevalence and geographical distribution of these parasites in central Germany, a total of 569 foxes were examined by dissection.

**Methods:**

Pluck (heart and lung) and faecal samples of red foxes were collected from three regions of Germany. Lungs, hearts and adjacent vessels were processed for adult nematode detection. Parasitological diagnoses of faecal samples were performed by SAF technique, *Giardia*- and *Cryptosporidium*-Coproantigen-ELISAs and by a duplex copro-PCR for the detection of *A. vasorum* and *C. vulpis* DNA.

**Results:**

Foxes originated from three Federal States of central Germany: Thuringia (*n* = 359); Rhineland-Palatinate (*n* = 121) and Hesse (*n* = 89). High prevalences for all three nematodes were detected, with *E. aerophilus* (69.4%; 395/569), followed by *C. vulpis* (32.3%; 184/569) and *A. vasorum* (14.1%; 80/569). In case of *A. vasorum*, prevalences varied significantly between Federal States, with the highest prevalence of 27.3% in Rhineland-Palatinate, followed by 19.1% and 8.4% in Hesse and Thuringia, respectively. The presence of *A. vasorum* in fox populations showed a rather patchy distribution, increasing from north-eastern to south-western regions. Analyses on *C. vulpis* revealed prevalences of 35.1%, 30.3% and 25.6% (Thuringia, Hesse and Rhineland-Palatinate, respectively). The most prevalent lungworm nematode was *E. aerophilus*, with a prevalence of 75.2%, 71.9% and 66.9% (Rhineland-Palatinate, Hesse and Thuringia, respectively) and an almost area-wide equal distribution. Significant differences for single parasite prevalences within geographical regions of the Federal States could be detected whilst no correlation between age or gender and parasite occurrence was estimated. Weak seasonality for the winter months for *A. vasorum*, stronger correlation to spring and late summer for *C. vulpis* and no correlation to any season for *E. aerophilus* were detected. The method of dissection revealed a significantly higher sensitivity for *C. vulpis* when compared with the results of the duplex copro-PCR.

**Conclusions:**

A sylvatic cycle was confirmed for all three lungworm nematodes in the examined area. The prevalences for all three lungworm nematodes are some of the highest recorded so far in German foxes. The data suggest that *A. vasorum* might be spreading from south-western to north-eastern parts of Germany.

**Electronic supplementary material:**

The online version of this article (10.1186/s13071-018-2672-4) contains supplementary material, which is available to authorized users.

## Background

The nematodes *Angiostrongylus vasorum*, *Crenosoma vulpis* and *Eucoleus aerophilus* (syn. *Capillaria aerophila*) are the most important lungworm species infecting wild and domestic canids in Europe [1, 2]. Within the last years, they have become the focus of increased attention from the scientific community due to their occurrence in previously unreported regions in several European countries [2]. *Angiostrongylus vasorum* has been found in red foxes (*Vulpes vulpes*) [3], dogs [4–6], wolves [7], coyotes [8] and even mustelid species [9]. The distribution of the parasite is rather patchy, with ‘hyperendemic’ geographical regions being surrounded by areas in which the prevalence of *A. vasorum-*positive animals is rather low [3, 10].

The fox lungworm *C. vulpis* has also been reported in raccoon dogs [11], wolves [12] and coyotes [13]. *Crenosoma vulpis* is distributed in several European countries as well as in Alaska and in the Atlantic site of Canada [14]. The distribution in Germany has a rather extensive character and is not focally restricted, but equally distributed [6, 15].

The nematode *E. aerophilus* has been reported in foxes [11, 16–18], but also in racoon dogs [11], hedgehogs [19], wolves [12], lynxes [20], mustelids [21], as well as in cats [22] and in domestic dogs [22]. The parasite has been reported from several European and American countries [2, 22–26], but no clear distribution tendencies - as for *A. vasorum* - are described so far.

For *A. vasorum*, which is the most pathogenic of the three species, clinical signs may vary from mild, such as coughing or exercise intolerance, to signs of a fatal cardiopulmonary disease (reviewed in [27, 28]) and, furthermore haemorrhagic disorders [4] and even nervous complaints [29]. Similar to domestic dogs, pathology has also been observed in *A. vasorum-*infected foxes [30–32], but the effect of this nematode on fox health and population dynamics still remains uncertain [3]. It is widely suggested that foxes act as reservoir hosts of domestic dog infections [3, 33], although the relative contribution of larvae shed by dogs or foxes to canine infections has not yet been completely determined [3]. According to Morgan et al. [3], the dynamics of parasite transmission across the wild-domestic animal boundary is likely to be influenced by a wide range of factors. Here for example, high fox population densities in areas of human habitation are discussed, even though an increased *A. vasorum* transmission from foxes to dogs is not necessarily evidenced for other parasites, such as the tapeworm *Echinococcus multilocularis*, and its associated rise in the incidence of human disease [34]. Recent geographical spatial reports on canine angiostrongylosis in Germany evidence that *A. vasorum* is potentially expanding its geographical range from its original occurrence in southern and western parts of Germany [6] to the North and East [35]. A recent nationwide epidemiological survey [36], including > 12,000 domestic dogs, on the distribution and risk factors of angiostrongylosis and crenosomosis confirmed these geographical expanding tendencies.

In the past, *A. vasorum*-infected foxes were frequently found in close proximity to foci of canine angiostrongylosis [3, 30], but in the absence of genetic data, estimates of the degree of overlap in parasite populations between definitive host populations can only be speculative [1]. For *E. aerophilus*, first genetic analyses illustrated that some sub-populations of the parasite in fact co-infect pets and wildlife [37].

The current study aims to determine actual lungworm prevalences in fox populations in central Germany to better understand the complex epidemiology of these diseases.

## Methods

### Animals and sample collection

From November 2011 to February 2013, carcasses of red foxes (*V. vulpes*) (*n* = 569) killed by hunters, road accidents, for monitoring reasons or simply found dead in different counties of three central Federal States of Germany (Hesse, Rhineland-Palatinate and Thuringia) were subjected to necropsies at the regional Veterinary Authorities within the Federal States. Information on the find spot and the date of collection were recorded, wherever applicable. The carcasses were collected within 1–3 days after killing. This might not be applicable for foxes having died by road accidents or simply found dead. All fox carcasses were kept frozen at -80 °C for at least 48 h to inactivate *Echinococcus* eggs. The animals were classified by gender and age [young (approximately up to 2 years): if incisors were clean and unworn; adult (over 2 years): if incisors showed loss of lobulation and brown dentin spots on occlusal surfaces]. At necropsy, heart and lungs were isolated from the carcasses, taking care not to rupture the pulmonary trunk.

### Parasite isolation from the pulmonary trunk and parasite identification

After defrosting, the heart, pulmonary arteries and further pulmonary vessels, trachea, bronchi and larger bronchioles were opened via incision. The heart was separated from the lungs by transecting the major blood vessel as close as possible to the lungs, and all ventricles as well as the pulmonary arterial trunk were opened and inspected visually for nematodes. Both heart auricles and ventricles were incised transversally between the base and the apex of the heart. Blood clots were dissolved by digital pressure and the heart chambers were rinsed with tap water through a sieve of 150 μm pore size. The trachea was opened longitudinally and inspected carefully for nematodes. The lung lobes were dissected by opening all visible large pulmonary vessels down to the narrowest practicable point (*c.*1 mm diameter). All detected nematodes were collected and further identified (see below). Afterwards, bronchi were likewise dissected and then rinsed with tap water over a sieve of 150 μm aperture. Then, water was pumped vigorously through the lung tissues using tap pressure, and flushing was continued until lungs appeared pale and devoid of blood and the washing fluid appeared clear. All washings were sieved. The residues of each sieve were resuspended in tap water and examined under a binocular dissecting microscope (Leica MZ 75, Wetzlar, Germany) using 0.63 × 10.0× magnification for nematodes, larvae or eggs. The nematodes were further identified using a light microscope (Olympus BH-2, Hamburg, Germany) equipped with a digital camera (Olympus SC30, Hamburg, Germany). The parasites were characterized morphologically as described elsewhere [38]. Adult female and male nematodes of all species found were washed with sterile phosphate-buffered saline (PBS) before freezing at -80 °C.

### Copro-PCR for simultaneous detection of *A. vasorum* and *C. vulpis* DNA

After defrosting of the fox carcasses, faecal samples were taken from the rectal content (565/569 foxes). In four foxes (three from Thuringia and one from Hesse) the rectum was missing, which was the reason why these foxes could not be examined by copro-PCR. Copro-DNA was isolated from 1 g faeces according to Nunes et al. [39] performing a combination of homogenization by horizontal vortexing with glass beads (Ø 4 mm, Carl Roth, Karlsruhe, Germany; 30 beads/sample; Vortex-Genie 2 with the MoBio horizontal 15 ml tube holder adapter, 10 min vortexing) and an extraction protocol using a commercial kit (QIAamp DNA stool-kit, protocol for larger volumes, Qiagen, Hilden, Germany).

For *C. vulpis* a diagnostic real-time PCR based on the ribosomal DNA of the internal transcribed spacer 2 (ITS2) sequence was designed. Only two sequences of *C. vulpis* (18S and 28S ribosomal RNA gene) were available in GenBank. These sequences are highly conserved among different genera of metastrongyloid nematodes making them less suitable for species-specific PCR assays. Therefore, the more variable ITS regions of the ribosomal RNA genes using the universal nematode forward primer NC16 (5′-AGT TCA ATC GCA ATG GCT T-3′ [40]) and reverse primer NC2 (5′-TTA GTT TCT TTT CCT CCG CT-3′ [41]) were amplified and sequenced (GenBank: KF836608). A probe-based real-time PCR assay was designed with Beacon Designer 8.01 (Premier Biosoft, Palo Alto, USA). The specificity of primers and probe was verified by BLAST analysis of the GenBank database and with DNA isolated from *C. vulpis* and *A. vasorum*. This assay was combined with the *A*. *vasorum* real-time PCR [42] as duplex PCR. The efficiency of the duplex PCR - determined by the standard curve method of 1:10 dilutions of *A. vasorum* and *C. vulpis* genomic DNA in a background of DNA from a negative fox faecal sample - was 100% for *A. vasorum* and 83% for *C. vulpis* (not shown).

The duplex real-time PCR for the detection of *A. vasorum* and *C. vulpis* DNA used the following primers and probes: forward primer I2F10 (5′-CGC ATG ATG AAA GAA TGC TG-3′), reverse primer I2R9 (5′-GAC GAC GAC GAC AAC CAC T-3′) and probe I2P2 (FAM-ACA ACA TTG CTT GTC GAA CGG CGT T-BHQ1) according to Jefferies et al. [42] for the detection of *A. vasorum* DNA and forward primer CvITS2f (5′-GCA TGA TAT TCG ACG ATT G-3′), reverse primer CvITS2r (5′-GTG TGA TCT AGT CAT GTA TAA C-3′) and probe CvITS2p (HEX-CAG CAA TGA GAA GAC ACT ATA CAC AAG-BHQ1) for the detection of *C. vulpis* DNA. For PCR analyses the following mastermix was used: 500 nM of each primer, 200 nM of each probe, 10 μl peqGOLD Hot Start-Mix Real-Time (2×, Peqlab, Erlangen, Germany), 2 μl DNA (sample or positive control), 0.2 μl BSA (10 mg/ml) and distilled water adjusted to 20 μl. The PCR was performed, using the following conditions: 1 × 95 °C 5 min, 45 × 94 °C 15 s and 60 °C 1 min with fluorescence detection in the green (FAM) and yellow channel (HEX). For positive controls in run to run comparison a PCR-negative fox faecal sample was spiked with *A. vasorum* and *C. vulpis* DNA at two concentrations (500 and 50 pg/μl). A Ct of ≤ 40 was regarded as positive.

### Statistical analysis

The statistical analysis of the data was performed with the help of the validated statistical programme packages BMDP/Dynamic, Release 8.1 [43] and BiAS [44]. The presence of nematodes or DNA detection during PCR for every variable was dichotomised into negative (= not present) and positive (= present) to calculate the estimated prevalences and their corresponding 95% confidence intervals (CI).

In a first step of the analysis the raw association between the presence of the three nematode species and the potential epidemiological influencing factors ‘surveyed region’ resp. ‘geographical area within the surveyed region’, ‘gender’, ‘age (young or adult)’ and ‘date of hunting resp. detection (both: monthly and quarterly)’ was analysed using Pearson’s chi-square test for contingency-tables. For these global comparisons differences were considered as significant at a level of *P* ≤ 0.05.

In the case of statistically significant global differences, pairwise group comparisons followed with either the chi-square test or Fisher’s exact test, controlling the global type I error rate using the Bonferroni-Holm-procedure.

Within the single Federal States the categorization into governmental districts (Darmstadt, Kassel, Giessen for Hesse) or geographical areas (North, Central, South for Rhineland-Palatinate and North, West, East, Central for Thuringia) was performed and the prevalence of the three nematode species was compared in just the same manner.

In a second step of the analysis a multi-way logistic regression model was applied (programme BMDPLR) to consider the possible overlay effects of the independent variables region, gender, age and date. The dependent variables were built by the presence of the examined nematodes (present or absent). In this way adjusted regression coefficients and odds ratios (OR) could be computed. The number of samples being analysed via the multiple logistic regression model had to be reduced to *n* = 192, because this method needs complete observations, which was not the case for all samples.

In the third part of the analysis the association between the common occurrence of the different nematodes was analyzed. This was done by cross-tabulation, the computation of the kappa-coefficient (κ) as a measure of concordance and computing the chi-square test.

### Geographical information system (GIS) database and data visualisation

The data were analysed by a GIS-based approach using the program RegioGraph 10 (GfK GeoMarketing, Bruchsal, Germany) to visualise the regional distribution of collected and analysed fox carcasses and positive results of the necropsies for the three different nematodes on administrative maps. For Hesse and Rhineland-Palatinate the place of hunting or discovery was associated to the corresponding hunting ground with its postal code. For Thuringia the place of hunting or discovery was directly associated to the corresponding postal code. Using these postal codes as points of reference prevalences were displayed on maps with administrative and postcode boundaries as differently coloured areas.

## Results

### Nematode burden and co-infections

In all three nematode populations found in the hearts and lungs of foxes, both genders were present and gravid female nematodes carrying eggs were frequently detected. Worm burdens ranging between 1–39 (*A. vasorum*), 1–114 (*C. vulpis*) and 1–95 (*E. aerophilus*) were recorded, varying greatly between the nematode species (see Table 1 for details). In total, 440 out of 569 foxes were positive for lungworms.

**Table 1:** Worm burdens in the cardiopulmonary tract

Nematode species	Intensity^a^			
	Median	Lower quartile	Upper quartile	Range
*Angiostrongylus vasorum*	2	1	3	1–39
*Crenosoma vulpis*	4	1	9	1–114
*Eucoleus aerophilus*	5	2	14	1–95

^a^Negative individuals not included

**Fig. 1:**
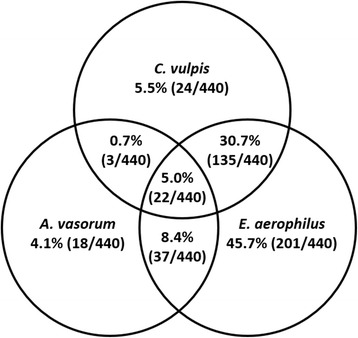
Mono-, double and triple infections with *Angiostrongylus vasorum*, *Crenosoma vulpis* and *Eucoleus aerophilus*, percentage based on the total number of lungworm-positive fox carcasses (*n* = 440)

The highest number of mono-infections was recorded for *E. aerophilus* (45.7%; 201/440), followed by double infections of *E. aerophilus* and *C. vulpis* (30.7%; 135/440). For a detailed overview including all recorded double and triple infections see Fig. 1.

Statistical analysis of association revealed a weak correlation of *E. aerophilus* and *C. vulpis* double infections (kappa-coefficient *κ* = 0.181) with high statistical significance (Pearson’s chi-square test: *χ*^2^ = 32.41, *df* = 1, *P* <  0.0001). No significant association could be detected for double infections of *A. vasorum* and *C. vulpis* (*κ* = -0.008, *P* = 0.883) or *A. vasorum* and *E. aerophilus* (*κ* = 0.019; *P* = 0.365).

### Lungworm prevalences and geographical distribution

Lungworm nematodes were found in 77.3% (*n* = 440; 95% CI: 73.7–80.7%) of all 569 analyzed foxes. The overall prevalences of *A. vasorum*, *C. vulpis* and *E. aerophilus* in foxes were 14.1% (*n* = 80; 95% CI: 11.3–17.2%), 32.3% (*n* = 184; 95% CI: 28.5–36.4%) and 69.4% (*n* = 395; 95% CI: 65.5–73.2%), respectively (see Table 2). The respective prevalences differed significantly from each other as detected by McNemar’s test of symmetry (*P* <  0.0001 for all comparisons). Only the occurrence of *E. aerophilus* and *C. vulpis* infections showed a statistically significant positive association (*κ* = 0.181; Pearson’s chi-square test: *χ*^2^ = 32.41, *df* = 1, *P* <  0.0001).

**Table 2:** Distribution of *Angiostrongylus vasorum* (Av), *Crenosoma vulpis* (Cv) and *Eucoleus aerophilus* (Ea) positive carcasses per Federal State and in total (percentage, total number and 95% confidence interval)

Federal state	Percentage Av positive in total (x/y) [95% CI]	Percentage Cv positive in total (x/y) [95% CI]	Percentage Ea positive in total (x/y) [95% CI]	Percentage Av + Cv positive (x/y) [95% CI]	Percentage Av + Ea positive (x/y) [95% CI]	Percentage Cv + Ea positive (x/y) [95% CI]	Percentage Av + Cv + Ea positive (x/y) [95% CI]
Hesse	19.1 (17/89) [11.5–28.8]	30.3 (27/89) [21.0–41.0]	71.9 (64/89) [61.4–80.9]	1.1 (1/89) [0.0–6.1]	11.2 (10/89) [5.5–19.7]	23.6 (21/89) [15.2–33.8]	4.5 (4/89) [1.2–11.1]
Rhineland-Palatinate	27.3 (33/121) [19.6–36.1]	25.6 (31/121) [18.1–34.4]	75.2 (91/121) [66.5–82.6]	0.8 (1/121) [0.0–4.5]	11.6 (14/121) [6.5–18.7]	15.7 (19/121) [9.7–23.4]	8.3 (10/121) [4.0–14.7]
Thuringia	8.4 (30/359) [5.7–11.7]	35.1 (126/359) [30.1–40.3]	66.9 (240/359) [61.7–71.7]	0.3 (1/359) [0.0–1.5]	3.6 (13/359) [1.9–6.1]	26.5 (95/359) [22.0–31.3]	2.2 (8/359) [1.0–4.3]
Total	14.1 (80/569) [11.3–17.2]	32.3 (184/569) [28.5–36.4]	69.4 (395/569) [65.5–73.2]	0.5 (3/569) [0.1–1.5]	6.5 (37/569) [4.6–8.9]	23.7 (135/569) [20.3–27.4]	3.9 (22/569) [2.4–5.8]

*Abbreviations*: *CI* confidence interval, *x* fox carcasses positive for a specific parasite, *y* total number of foxes examined per federal state and in total

**Fig. 2:**
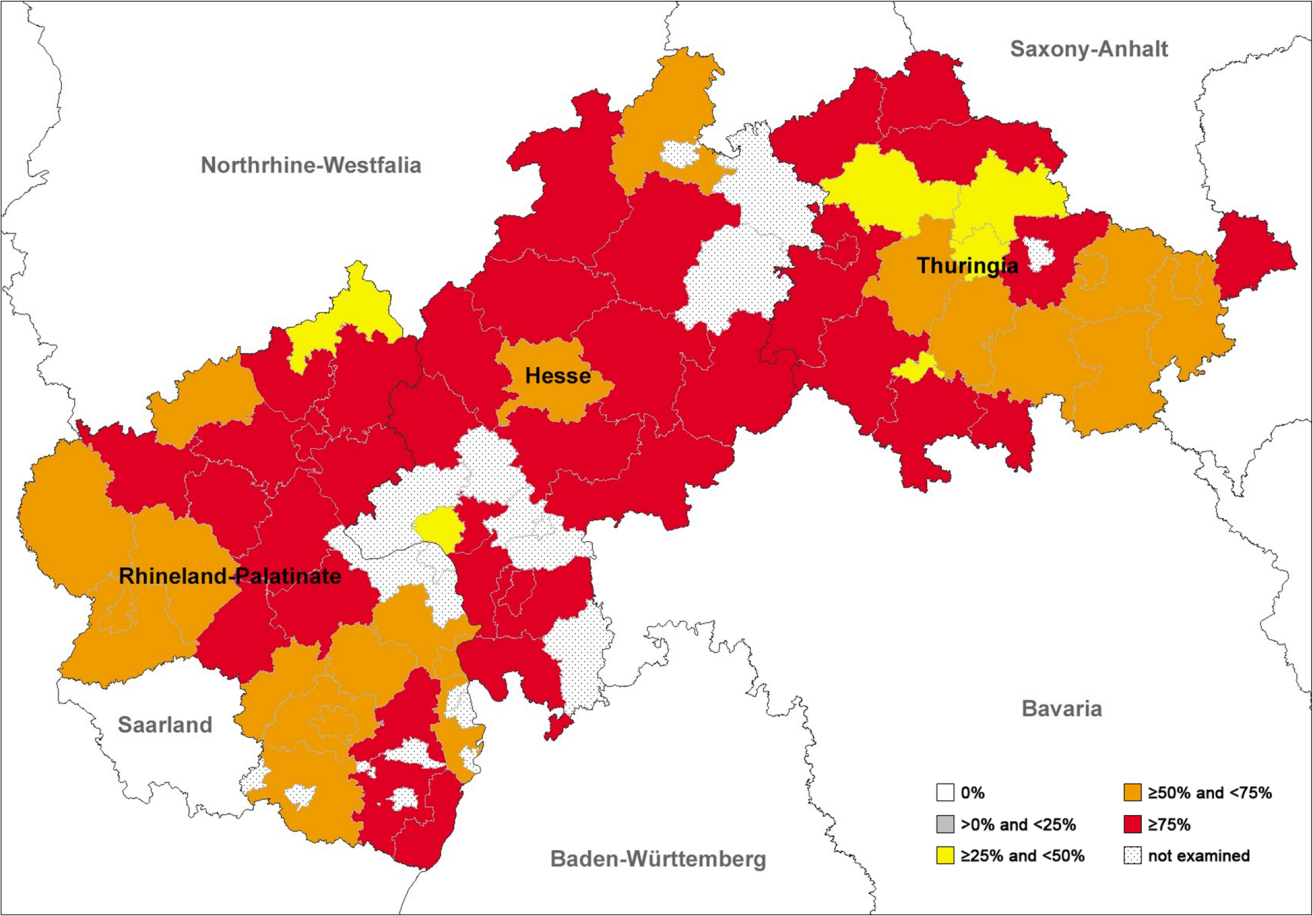
Prevalence distribution of *Eucoleus aerophilus* detected by dissection of 569 foxes from central Germany

**Fig. 3:**
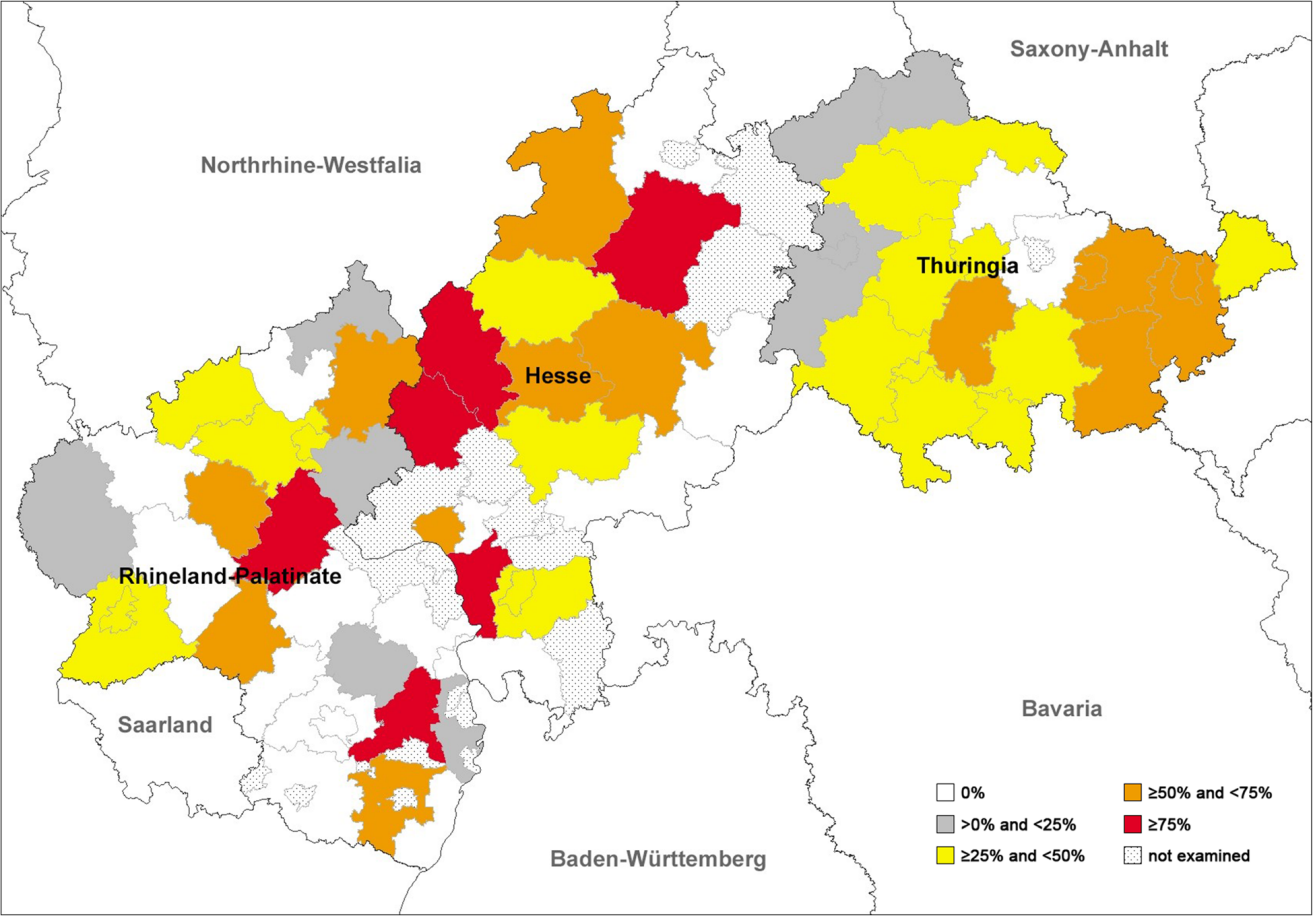
Prevalence distribution of *Crenosoma vulpis* detected by dissection of 569 foxes from central Germany

**Fig. 4:**
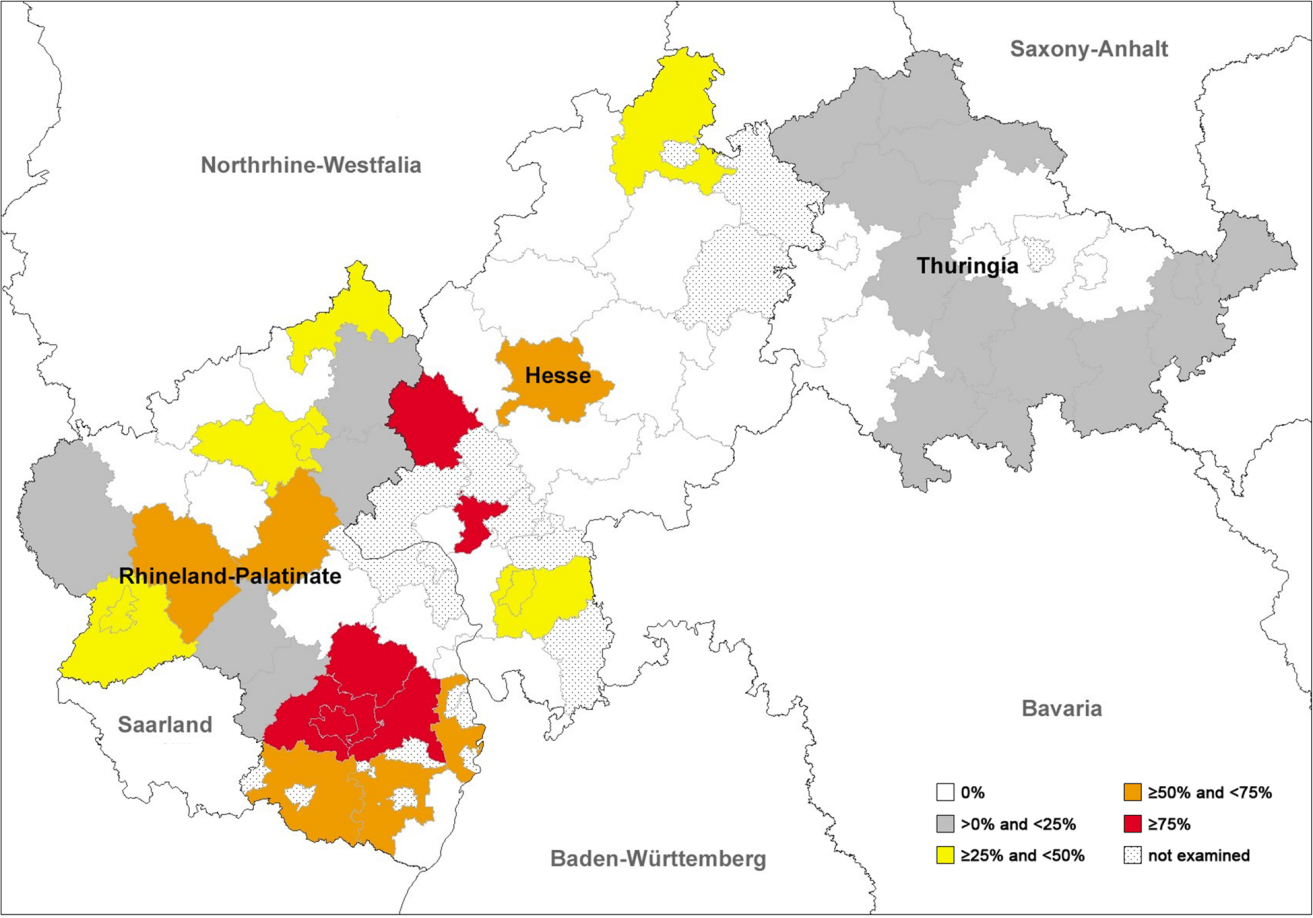
Prevalence distribution of *Angiostrongylus vasorum* detected by dissection of 569 foxes from central Germany

Sampling areas coloured differently into regions in which positive foxes have been detected as well as regions with exclusively negative foxes are shown on administrative maps in Figs. 2, 3 and 4. Prevalences for the three nematodes in the three Federal States are listed in Table 2. Chi-square tests revealed that the prevalences of *A. vasorum* between the Federal States were statistically significantly different (*χ*^2^ = 29.019, *df* = 2, *P* <  0.0001). The highest prevalence was found in Rhineland-Palatinate (27.3%) followed by Hesse (19.1%) and Thuringia (8.4%).

The number of positive samples per county or city and per governmental district or geographical region in the Federal States is depicted in Additional files 1, 2 and 3: Tables S1, S2 and S3.

Statistical calculation on county/city basis was not performed due to too low fox numbers in some of the counties/cities. After categorization into larger regions (see also Additional files 1, 2 and 3: Tables S1, S2 and S3) significant differences could be observed in the occurrence of all three nematodes as detailed below.

For *A. vasorum* significant global differences were detected within Rhineland-Palatinate (Pearson’s chi-square test, *χ*^2^ = 22.901, *df* = 2, *P* <  0.0001; Table 3). This could be specified to differences between southern and northern (*χ*^2^ = 14.855, *df* = 1, *P-Bonf.-Holm* = 0.0002) as well as southern and central areas (*χ*^2^ = 17.025, *df* = 1, *P-Bonf.-Holm* <  0.0001), with southern areas generally showing higher prevalences (59.4%). Furthermore, significant global differences were detected in Thuringia (*χ*^2^ = 8.378, *df* = 3, *P* = 0.039), especially between northern and western (*χ*^2^ = 7.904, *df* = 1, *P-Bonf.-Holm* = 0.029) areas, with higher prevalences in the northern (14.1%) part. For Hesse no significant differences could be shown (*χ*^2^ = 3.767, *df* = 2, *P* = 0.152; Table 3).

**Table 3:** Significant differences between geographical regions within the three examined Federal States for *Angiostrongylus vasorum* (Av), *Crenosoma vulpis* (Cv) and *Eucoleus aerophilus* (Ea) positive fox carcasses

Federal state	Comparison geographical regions	*P*-value		
		Av^a^	Cv^a^	Ea^a^
Hesse	Global comparison	ns	0.001	ns
	Giessen *vs* Kassel	ns	0.0006	ns
	Darmstadt *vs* Kassel	ns	0.0115	ns
	Giessen *vs* Darmstadt	ns	ns	ns
Rhineland-Palatinate	Global comparison	< 0.0001	ns	ns
	South *vs* North	0.0002	ns	ns
	South *vs* Central	< 0.0001	ns	ns
	North *vs* Central	ns	ns	ns
Thuringia	Global comparison	0.039	0.0038	0.039
	North *vs* West	0.029	ns	0.071
	Central *vs* West	ns	ns	ns
	North *vs* East	ns	0.0036	ns
	North *vs* Central	ns	ns	ns
	East *vs* West	ns	0.044	ns
	East *vs* Central	ns	ns	ns

^a^For underlying prevalences see Additional files 1, 2 and 3: Tables S1, S2 and S3

*Abbreviation*: *ns* not significant

For *C. vulpis* significant differences were identified on the level of Hesse (global *P*-value = 0.001, *χ*^2^ = 13.767, *df* = 2; Table 3) between the governmental districts of Giessen and Kassel (*χ*^2^ = 13.904, *df* = 1, *P-Bonf.-Holm* = 0.0006) as well as Darmstadt and Kassel (*χ*^2^ = 7.722, *df* = 1, *P-Bonf.-Holm* = 0.011), with Giessen showing highest prevalence (58.8%), followed by Darmstadt (38.7%). For Thuringia significant differences were detected (global *P*-value = 0.0038, *χ*^2^ = 13.435, *df* = 3) especially between north and east (*χ*^2^ = 11.789, *df* = 1, *P-Bonf.-Holm* = 0.0036), and east and west (*χ*^2^ = 6.854, *df* = 1, *P-Bonf.-Holm* = 0.044), with highest prevalences in the eastern part of Thuringia (50.7%) (see Table 3). Statistically significant differences for the lungworm *E. aerophilus* are shown in Table 3.

Statistical comparison on the median numbers of worms found in lungworm-positive samples (worm burden excluding negative samples) between the three Federal States revealed no statistically significant differences for *A. vasorum* and for *C. vulpis* by Kruskal-Wallis test (*H* = 1.70, *df* = 2, *P* = 0.43 and *H* = 0.38, *df* = 2, *P* = 0.83, respectively), while there were statistically significant differences for *E. aerophilus* between the investigated states (*H* = 12.45, *df* = 2, *P* = 0.0020). Pairwise comparison by the Dunn’s test (Bonferroni-Holm-corrected *P*-values) of the three Federal states regarding this lungworm species revealed significant differences between Hesse and Thuringia (*P*-*Bonf.-Holm* = 0.0039) and barely not significant differences between Rhineland-Palatinate and Thuringia (*P-Bonf.-Holm* = 0.060).

The same comparison between the regions within the Federal States revealed statistical significant differences only for *C. vulpis* in Thuringia (*H* = 10.46, *df* = 3, *P* = 0.015). Pairwise comparisons between the regions by means of the Dunn’s test showed an increased median in the western region of this state in contrast to the northern region (*P-Bonf.-Holm* = 0.008).

Comparing the absence of positive samples in the different counties/cities, altogether 25/58 counties/cities (11 Hessian, 7 Rhineland-Palatinian and 7 Thuringian) were found with a negative result for *A. vasorum*. Sixteen out of 58 (5 Hessian, 9 Rhineland-Palatinian and 2 Thuringian) counties/cities were negative for *C. vulpis* and none of the examined counties/cities were negative for *E. aerophilus*, indicating an almost equal, area-wide distribution of *E. aerophilus* (see also Fig. 2), a slightly less area-wide, but still widespread distribution of *C. vulpis* (see also Fig. 3) and a focal, patchy distribution of *A. vasorum* (see also Fig. 4) in the examined area.

### Seasonal distribution and further epidemiological parameters of lungworm-positive fox carcasses

Foxes were collected from November 2011 to February 2013. During this time span, all three lungworm species were detected in foxes all year round, even though the sample numbers were varying within the time span. Only from Thuringia were fox carcasses available during the entire sampling period. For the number of positive samples per season, gender and age see Table 4.

**Table 4:** Distribution of *Angiostrongylus vasorum* (Av), *Crenosoma vulpis* (Cv)and *Eucoleus aerophilus* (Ea) positive fox carcasses in total over the time span (November 2011 - February 2013) divided by season, gender, and age

Grouping variable	Group name	Percentage Av positive in total (x/y)	Percentage Cv positive in total (x/y)	Percentage Ea positive in total (x/y)
Season	Winter	19.4 (19/98)	22.4 (22/98)	78.6 (77/98)
	Spring	8.7 (9/103)	34.0 (35/103)	61.2 (63/103)
	Summer	10.4 (19/182)	46.7 (85/182)	70.9 (129/182)
	Autumn	18.1 (33/182)	23.1 (42/182)	67.0 (122/182)
	*P*-value	0.028	< 0.0001	0.0503
Gender	Male	21.8 (34/156))	30.8 (48/156)	78.2 (122/156)
	Female	20.2 (24/119)	29.4 (35/119)	65.5 (78/119)
	*P*-value	0.743	0.808	0.0195
Age	Juvenile	20.0 (14/70)	25.7 (18/70)	80.0 (56/70)
	Adult	24.5 (36/147)	27.9 (41/147)	72.8 (107/147)
	*P*-value	0.463	0.736	0.251

*Abbreviations*: *x* fox carcasses positive for a specific parasite, *y* total number of foxes examined

Furthermore, raw prevalence differences between genders could be shown only for *E. aerophilus* (*χ*^2^ = 5.454 *df* = 1, *P* = 0.0195; Table 4). Thus, male foxes (78.2%) were more often infected than female ones (65.5%). Referring to age groups, no statistically significant differences were detected for any lungworm species.

Statistical comparison for the single parasites between the seasons (four quarters per year) revealed a significant difference for *A. vasorum* (chi-square test, *χ*^2^ = 9.13, *df* = 3, *P* = 0.028) and for *C. vulpis* infections (chi-square test, *χ*^2^ = 28.687, *df* = 3, *P* < 0.0001). Thus, *A. vasorum* infections were more often detected in autumn (18.1%) and winter (19.4%) season while the highest *C. vulpis* prevalences were found in summer (46.7%) (see Table 4). For *E. aerophilus* the differences were at the margin of significance (*P* = 0.0503).

To take into account that there are possible overlapping effects between the considered epidemiological parameters, a multiple logistic regression model was fitted to the data. However, complete observations on all criteria were not available for all foxes (reduced sample size: *n* = 192). This resulted in weaker indications of significance when compared to analyses on raw prevalence differences. Nonetheless, significant seasonal differences in prevalences were still observed for *A. vasorum* (*F* = 3.80, *df* = 3, *P* = 0.0112) and *C. vulpis* (*F* = 6.88, *df* = 3, *P* = 0.0002) infections as well as differences between the Federal States for *A. vasorum* (*F* = 3.07, *df* = 2, *P* = 0.049).

In this multiple analysis the formerly found prevalence differences for *A. vasorum* between spring and late summer in comparison to the winter months could be confirmed in the form of low odds ratios (OR): OR (spring to winter) = 0.21 (95% CI: 0.05–0.82); OR (early summer to winter) = 0.24 (95% CI: 0.08–0.75). In contrast, for *C. vulpis* higher odds ratios (directly connected with prevalences) in spring and late summer in relation to the winter months were detected: OR (spring to winter) = 5.9 (95% CI: 1.54–22.3); OR (early summer to winter) = 12.8 (95% CI: 3.61–45.1).

### Detection of *A. vasorum* and *C. vulpis* DNA in fox faeces via duplex PCR

Rectally obtained faecal material from 565 foxes was examined by copro-PCR. Overall, DNA of *A. vasorum* and *C. vulpis* was detected in 65 out of 565 (11.5%; 95% CI: 9.0–14.4%) and 95 out of 565 (16.8%; 95% CI: 13.8–20.2%) faecal DNA samples, respectively, via duplex real-time PCR. Six samples (1.1%; 95% CI: 0.4–2.3%) showed a double infection with both lungworms (*A. vasorum* and *C. vulpis*). Matches and discrepancies comparing the results of the copro-PCR with that of the dissection are depicted in Table 5.

**Table 5:** Comparison of dissection and copro-PCR as diagnostic means for the detection of *Angiostrongylus vasorum* and *Crenosoma vulpis* in foxes: total number (percentage) [95% confidence interval]

	*A. vasorum*			*C. vulpis*		
	Copro-PCR		Total	Copro-PCR		Total
	+	–		+	–	
Dissection						
+	37 (6.6) [4.7–8.9]	43 (7.6) [5.6–10.1]	80	82 (14.5) [11.7–17.7]	102 (18.1) [15.0–21.5]	184
–	28 (5.0) [3.3–7.1]	457 (80.9) [77.4–84.0]	485	13 (2.3) [1.2–3.9]	368 (65.1) [61.0–69.1]	381
Total	65 (11.5) [9.0–14.4]	500 (88.5) [85.6–91.0]	565	95 (16.8) [13.8–20.2]	470 (83.2) [79.8–86.2]	565

## Discussion

*A. vasorum* is parasitizing the heart and pulmonary arteries, and has been documented in dogs in Germany with an endemic presence in certain areas [6, 35, 45]. Western Federal States have been in the focus of canine examinations for *A. vasorum* infections [35, 45] with prevalences between 0.3% and 7.4%. The whole of Germany has been the focus of a recent GIS-supported epidemiological analysis on canine angiostrongylosis/crenosomosis investigating in total 12,682 dogs with positive samples mainly originating from western and southern areas and showing prevalences of 2.3% (*A. vasorum*) and 2.2% (*C. vulpis*) [36]. The aim of the underlying study was to gain data on lungworm infections in red foxes from Germany, with a focus on endemic areas of canine lungworm infections. Past German wild reservoir studies with sample sizes between 100 and 400 foxes reported no *A. vasorum* occurrence, a maximum prevalence of 35% for *C. vulpis* and up to 77.8% prevalence for *E. aerophilus* [17, 46–49], while a recent study examining foxes in Brandenburg (eastern Germany) detected 9.0% prevalence for *A. vasorum* via PCR in lung tissue [50]. Thus, the overall prevalence of the current study for *A. vasorum* (14.1%) is the highest reported so far for foxes in Germany. The prevalences for *C. vulpis* (32.3%) and *E. aerophilus* (69.4%) are some of the highest reported so far for foxes in Germany.

On a European level the current *A. vasorum* prevalence was comparable, e.g. to data obtained from some southern European countries [51, 52], but higher than data in some of the direct European neighbour countries [53–55]. Broken down onto the basis of the three Federal States (Thuringia: 8.4%; Hesse: 19.1%; Rhineland-Palatinate: 27.3%) the prevalence of *A. vasorum* in the last one was already comparable to other highly endemic regions, even though it does not reach Danish levels (48.6%) [56]. Concerning the *C. vulpis* data, this is one of the highest prevalence reported so far for Europe, surmounted only by data from a few European countries [11, 16, 57–61]. Prevalence of *E. aerophilus* is higher than in most of the previous European studies for foxes and comparable to numbers detected in the Netherlands [54], Hungary [60] and Denmark [56].

Based on our data, a sylvatic cycle can be assumed for all three lung nematodes. The importance of these cycles for the epidemiology in dogs cannot be judged by the actual data alone, but the study helps to fill a gap within the whole epidemiological context of lungworm infections in Germany. As recorded in other studies, prevalences in dogs are generally lower than the ones found in foxes [6, 22, 35, 45, 62, 63]. These findings could be linked to differences of host innate/adaptive immune reactions of foxes and domestic dogs against the same parasites. Consistently, foxes develop a variable and non-protective immunity against *A. vasorum*, and this might explain parasite tolerance and long-term survival in these wild canids [64].

Concerning the seasonal distribution of the three lungworms, a highly significant occurrence in summer was detected for *C. vulpis*, whilst *A. vasorum* occurrence showed the highest levels in the autumn and winter months. These findings could be linked to differences observed in the biology of the different intermediate host species (i.e. *Arion ater*, *A. distinctus*, *Tandonia sowerbyi* and Limacidae species) [65], particularly on their innate immune response in form of haemocyte-derived extracellular traps against infective lungworm larvae to be involved in gastropod-parasite interactions [66]. Unfortunately, from our own investigations on dissected foxes, it cannot be deduced when natural infections occurred.

In the case of *E. aerophilus* infections, seasonal data revealed no statistical significance. Endogenous factors, such as prepatency period as well as exogenous factors (micro- and macroclimatic conditions, intermediate host species and slug/snail population dynamics), might additionally influence the seasonality of the parasites [6, 65]. There was no significant correlation between prevalences of *A. vasorum* and age of the foxes (young and adult), comparable to studies by Magi et al. [67], but also in contrast to studies [31, 56], which confirmed explicitly young foxes to be infected with *A. vasorum*. The same was true concerning the gender of foxes. No significant correlation was detected, as previously described by Magi et al. [67] and in opposite to studies by Saeed et al. [56] where more positive males were identified. The precise parasite burden is often not examined in lungworm-related studies due to the fact that faecal examination is used as proof of infection. Concerning methods for the detection of *A. vasorum* in the final host, Houpin et al. [68] recently indicated sensitivities of 84% for dissection and visual examination of plucks, 69% for nested PCR and 76% for an antigen detection test, thus showing the advantages of the dissection method.

The underlying study revealed parasite burdens of 1–39 for *A. vasorum*, 1–114 for *C. vulpis* and 1–95 for *E. aerophilus*. For *A. vasorum*, parasite burdens in dogs have been reported between 1 and 7 in Newfoundland and Labrador [69], and between 1 and 88, with a mean of 17.4 adults in Danish foxes [31]. Thus, the mean infection load for *A. vasorum* was rather low in the current study. Double and triple infections have already been recorded for the three lung nematodes [31, 67]. Apart from fox infections with *C. vulpis* and *E. aerophilus*, which showed a significant correlation to one another, no other parasite correlations were statistically significant.

From the 1960s onwards, *A. vasorum* infection in dogs and foxes was reported at an accelerating rate, and the parasite became established in areas other than the long-term known hyperendemic foci, such as Ireland [70], the UK [71] and Denmark [33]. Since then, there has been a series of new records from different countries in Europe, increasing reports on angiostrongylosis in endemic areas of Europe and North America [1, 69]. For Germany, the geographical dispersal of *A. vasorum* infections in dogs has been reported in a number of studies, demonstrating the highest prevalences for southern and south-western regions [6, 35, 36, 45]. The prevalence of *A. vasorum* in definitive hosts is strongly affected by different factors, such as host density, parasite aggregation and sample structure, while microclimatic factors rather influence the intermediate host abundance and activity [72]. An expansion of canine *A. vasorum* endemic areas and/or emergence of this parasite in regions previously believed to be free was recently also reported for Belgium, Bulgaria, Great Britain, the Netherlands, Portugal, regions of Scandinavia or Slovakia [73–79].

For *C. vulpis* and *E. aerophilus*, the distribution pattern in foxes seems to be more generalized in Germany since similar prevalences of 25.6% and 75.2% (Rhineland-Palatinate), 30.3% and 71.9% (Hesse) and 35.1%and 66.9% (Thuringia) were found in the three examined Federal States, respectively. In line with these data, *C. vulpis* infections in dogs have been described with rather equal, but regionally piled areas [35].

The current geographical dispersal as well as the pattern of dispersion of *A. vasorum* and *C. vulpis* in foxes is also mirrored in dogs [6, 35, 45] with increasing tendencies from northeast to southwest [36]. The reason why the nematodes show significant differences in prevalence although having similar requirements in their life-cycle (same intermediate hosts) could be linked to multifactorial influences (e.g. early innate host immune reactions) and cannot be explained in detail by this study.

However, the actual spectrum of intermediate hosts may influence the epidemiology of these parasites as previously shown for *C. vulpis* [80]. In addition, the different microclimatic conditions and the cold hardiness of the first larvae of *C. vulpis* [56] may be of importance here.

Overall, *E. aerophilus* showed an almost area-wide, equal distribution with rather stable, high prevalences, as also reported for Great Britain [75] and Hungary [81].

Finally we analysed the value of a biomolecular approach for the detection of *A. vasorum* and *C. vulpis* in comparison to the dissection technique. Overall, the duplex copro-PCR reliably detected DNA of *A. vasorum* and *C. vulpis* in fox faeces without prior larval concentration. However, comparing on prevalence level, the dissection results outweighed those of the PCR, demonstrating a lower sensitivity for the copro-PCR. False negative and positive PCR results for the detection of *A. vasorum*-DNA in faecal samples have also been reported by others [42]. Besides intermittent larval shedding [82] other factors, such as the infection stage, the sample size, the DNA extraction method, the quality of the clinical samples, but also competitive inhibition by other DNAs or the presence of DNases in each faecal sample or inefficient cell lysis during the extraction process were discussed as basis of PCR failure [42].

## Conclusions

In conclusion, this study presents an overview on the occurrence of three important canid lungworm nematodes in a wide area and a large fox population in Germany. It thus provides essential epidemiological data from wildlife acting as natural reservoirs, which is urgently needed for the identification of endemic foci of canid lungworm infections and for predictions on the actual spread of *A. vasorum*. A sylvatic cycle could be here clearly confirmed for all three lungworm species investigated, with far higher prevalences than in the domestic canine cycle. A regional dispersal and prevalence increase from north-eastern to south-western areas, as observed in dogs (mainly for *A. vasorum* and *C. vulpis*), could also here be illustrated for German foxes. These data should provide new insights into the epidemiological situation of lungworm infections in free-ranging foxes and call for further investigations on lungworm infections associated to other wild canids (i.e. wolves) of Germany.

## Additional files

Additional file 1: Table S1. Distribution of *Angiostrongylus vasorum* (Av), *Crenosoma vulpis* (Cv) and *Eucoleus aerophilus* (Ea) positive carcasses per county/city in the Federal State of Hesse (percentage and total numbers). (DOCX 18 kb)

Additional file 2: Table S2. Distribution of *Angiostrongylus vasorum* (Av), *Crenosoma vulpis* (Cv) and *Eucoleus aerophilus* (Ea) positive carcasses per county/city in the Federal State of Rhineland-Palatinate (percentage and total numbers). (DOCX 17 kb)

Additional file 3: Table S3. Distribution of *Angiostrongylus vasorum* (Av), *Crenosoma vulpis* (Cv) and *Eucoleus aerophilus* (Ea) positive carcasses per county/city in the Federal State of Thuringia (percentage and total numbers). (DOCX 17 kb)

## Abbreviations

CI: Confidence interval
GIS: Geographical information system
PBS: Phosphate-buffered saline
PCR: Polymerase chain reaction

## References

[1] Morgan ER, Shaw SE, Brennan SF, De Waal TD, Jones BR, Mulcahy G (2005). *Angiostrongylus vasorum*: a real heartbreaker. Trends Parasitol.

[2] Traversa D, Di Cesare A, Conboy G (2010). Canine and feline cardiopulmonary parasitic nematodes in Europe: emerging and underestimated. Parasit Vectors.

[3] Morgan ER, Tomlinson A, Hunter S, Nichols T, Roberts E, Fox MT (2008). *Angiostrongylus vasorum* and *Eucoleus aerophilus* in foxes (*Vulpes vulpes*) in great Britain. Vet Parasitol.

[4] Chapman PS, Boag AK, Guitian J, Boswood A (2004). *Angiostrongylus vasorum* infection in 23 dogs (1999–2002). J Small Anim Pract.

[5] Willesen JL, Jensen AL, Kristensen AT, Koch J (2009). Haematological and biochemical changes in dogs naturally infected with *Angiostrongylus vasorum* before and after treatment. Vet J.

[6] Taubert A, Pantchev N, Vrhovec MG, Bauer C, Hermosilla C (2009). Lungworm infections *(Angiostrongylus vasorum*, *Crenosoma vulpis*, *Aelurostrongylus abstrusus*) in dogs and cats in Germany and Denmark in 2003-2007. Vet Parasitol.

[7] Hermosilla C, Kleinertz S, Silva LM, Hirzmann J, Huber D, Kusak J (2017). Protozoan and helminth parasite fauna of free-living Croatian wild wolves (*Canis lupus*) analyzed by scat collection. Vet Parasitol.

[8] Bourque A, Whitney H, Conboy G (2005). *Angiostrongylus vasorum* infection in a coyote (*Canis latrans*) from Newfoundland and Labrador, Canada. J Wildl Dis.

[9] Simpson VR, Tomlinson AJ, Stevenson K, JA ML, Benavides J, Dagleish MP (2016). A post-mortem study of respiratory disease in small mustelids in south-west England. BMC Vet Res.

[10] Kirk L, Limon G, Guitian FJ, Hermosilla C, Fox MT (2014). *Angiostrongylus vasorum* in great Britain: a nationwide postal questionnaire survey of veterinary practices. Vet Rec..

[11] Bružinskaitë-Schmidhalter R, Šarkûnas M, Malakauskas A, Mathis A, Torgerson PR, Deplazes P (2012). Helminths of red foxes (*Vulpes vulpes*) and raccoon dogs (*Nyctereutes procyonoides*) in Lithuania. Parasitology.

[12] Bagrade G, Kirjusina M, Vismanis K, Ozoliòs J (2009). Helminth parasites of the wolf *Canis lupus* from Latvia. J Helminthol.

[13] Bridger KE, Baggs EM, Finney-Crawley J (2009). Endoparasites of the coyote (*Canis latrans*), a recent migrant to insular Newfoundland. J Wildl Dis.

[14] Conboy G (2004). Natural infections of *Crenosoma vulpis* and *Angiostrongylus vasorum* in dogs in Atlantic Canada and their treatment with milbemycin oxime. Vet Rec..

[15] Barutzki D (2013). Nematode infections of the respiratory tract in dogs in Germany. Tieraerztl Prax Ausg K Kleintiere.

[16] Davidson RK, Gjerde B, Vikøren T, Lillehaug A, Handeland K (2006). Prevalence of *Trichinella* larvae and extra-intestinal nematodes in Norwegian red foxes (*Vulpes vulpes*). Vet Parasitol.

[17] Schöffel I, Schein E, Wittstadt U, Hentsche J (1991). Parasite fauna of red foxes in Berlin (west). Berl Münch Tieraerztl Wochenschr.

[18] Willingham AL, Ockens NW, CMO K, Monrad J (1996). A helminthological survey of wild red foxes (*Vulpes vulpes*) from the metropolitan area of Copenhagen. J Helminthol.

[19] Mizgajska-Wiktor H, Jarosz W, Piłacińska B, Dziemian S (2010). Helminths of hedgehogs, *Erinaceus europaeus* and *E. roumanicus* from Poznań region, Poland - coprological study. Wiad Parazytol.

[20] Szczesna J, Popiołek M, Schmidt K, Kowalczyk R (2008). Coprological study on helminth fauna in Eurasian lynx (*Lynx lynx*) from the Białowieza primeval Forest in eastern Poland. J Parasitol.

[21] Torres J, Miquel J, Fournier P, Fournier-Chambrillon C, Liberge M, Fons R (2008). Helminth communities of the autochthonous mustelids *Mustela lutreola* and *M. putorius* and the introduced *Mustela vison* in south-western France. J Helminthol.

[22] Traversa D, Di Cesare A, Milillo P, Iorio R, Otranto D (2009). Infection by *Eucoleus aerophilus* in dogs and cats: is another extra-intestinal parasitic nematode of pets emerging in Italy?. Res Vet Sci.

[23] Campbell BG, Little MD (1991). Identification of the eggs of a nematode (*Eucoleus boehmi*) from the nasal mucosa of north American dogs. J Am Vet Med Assoc.

[24] Conboy GA (2009). Helminth parasites of the canine and feline respiratory tract. Vet Clin N Am Small Anim Pract.

[25] Di Cesare A, Castagna G, Meloni S, Milillo P, Latrofa MS, Otranto D (2011). Canine and feline infections by cardiopulmonary nematodes in central and southern Italy. Parasitol Res.

[26] Mircean V, Titilincu A, Vasile C (2010). Prevalence of endoparasites in household cat (*Felis catus*) populations from Transylvania (Romania) and association with risk factors. Vet Parasitol.

[27] Bolt G, Monrad J, Koch J, Jensen AL (1994). Canine angiostrongylosis: a review. Vet Rec..

[28] Koch J, Willesen JL (2009). Canine pulmonary angiostrongylosis: an update. Vet J.

[29] Perry AW, Hertling R, Kennedy MJ (1991). Angiostrongylosis with disseminated larval infection associated with signs of ocular and nervous disease in an imported dog. Can Vet J.

[30] Simpson VR (1996). *Angiostrongylus vasorum* infection in foxes (*Vulpes vulpes*) in Cornwall. Vet Rec..

[31] Al-Sabi MN, Halasa T, Kapel CM (2014). Infections with cardiopulmonary and intestinal helminths and sarcoptic mange in red foxes from two different localities in Denmark. Acta Parasitol.

[32] Eleni C, Grifoni G, Di Egidio A, Meoli R, De Liberato C (2014). Pathological findings of *Angiostrongylus vasorum* infection in red foxes (*Vulpes vulpes*) from Central Italy, with the first report of a disseminated infection in this host species. Parasitol Res.

[33] Bolt G, Monrad J, Henriksen P, Dietz HH, Koch J, Bindseil E (1992). The fox (*Vulpes vulpes*) as a reservoir for canine angiostrongylosis in Denmark. Acta Vet Scand.

[34] Deplazes P, Hegglin D, Gloor S, Romig T (2004). Wilderness in the city: the urbanization of *Echinococcus multilocularis*. Trends Parasitol.

[35] Barutzki D, Schaper R (2009). Natural infections of *Angiostrongylus vasorum* and *Crenosoma vulpis* in dogs in Germany (2007–2009). Parasitol Res.

[36] Maksimov P, Hermosilla C, Taubert A, Staubach C, Sauter-Louis C, Conraths F (2017). GIS-supported epidemiological analysis on canine *Angiostrongylus vasorum* and *Crenosoma vulpis* infections in Germany. Parasit Vectors.

[37] Di Cesare A, Otranto D, Latrofa MS, Veronesi F, Perrucci S, Lalosevic D (2014). Genetic variability of *Eucoleus aerophilus* from domestic and wild hosts. Res Vet Sci.

[38] Georgi JR, Georgi ME (1991). Canine clinical parasitology.

[39] Nunes CM, Lima LG, Manoel CS, Pereira RN, Nakano MM, Garcia JF (2006). Fecal specimens preparation methods for PCR diagnosis of human taeniosis. Rev Inst Med Trop Sao Paulo.

[40] Chilton NB, Huby-Chilton F, Gasser RB (2003). First complete large subunit ribosomal RNA sequence and secondary structure for a parasitic nematode: phylogenetic and diagnostic implications. Mol Cell Probes.

[41] Gasser RB, Chilton NB, Hoste H, Beveridge I (1993). Rapid sequencing of rDNA from single worms and eggs of parasitic helminths. Nucleic Acids Res.

[42] Jefferies R, Morgan ER, Shaw SE (2009). A SYBR green real-time PCR assay for the detection of the nematode *Angiostrongylus vasorum* in definitive and intermediate hosts. Vet Parasitol.

[43] Dixon WJ (1993). BMDP statistical software manual. Volume 1 and 2.

[44] Ackermann H (2010). BiAS. für Windows, Biometrische Analyse von Stichproben, Version 9.08.

[45] Schnyder M, Schaper R, Bilbrough G, Morgan ER, Deplazes P (2013). Seroepidemiological survey for canine angiostrongylosis in dogs from Germany and the UK using combined detection of *Angiostrongylus vasorum* antigen and specific antibodies. Parasitology.

[46] Lamina J (1964). Der Parasitenbefall bei Rotfüchsen in Südhessen. Z Jagdwiss.

[47] Lucius R, Böckeler W, Pfeiffer AS (1988). Parasitic infestation of the domestic and wild animals of Schleswig-Holstein: parasites of the inner organs of the red fox (*Vulpes vulpes*). Z Jagdwiss.

[48] Nickel S, Hiepe T, Hansel U, Jurke E (1980). Parasites in the DDR. 5. The occurrence of helminths in the red fox (*Vulpes vulpes* L.). Angew Parasitol.

[49] Steinbach G, Welzel AV, Keyserlingk M, Stoye M (1994). On the helminthic fauna of the red fox (*Vulpes vulpes* L.) in southern lower Saxony. Z Jagdwiss.

[50] Härtwig V, Schulze C, Barutzki D, Schaper R, Daugschies A, Dyachenko V (2015). Detection of *Angiostrongylus vasorum* in red foxes (*Vulpes vulpes*) from Brandenburg, Germany. Parasitol Res.

[51] Eira C, Viganda J, Torres J, Miquel J (2006). The helminth community of the red fox, *Vulpes vulpes*, in Dunas de Mira (Portugal) and its effect on host condition. Wildl Biol Pract.

[52] Mañas S, Ferrer D, Castellà J, López-Martín JM (2005). Cardiopulmonary helminth parasites of red foxes (*Vulpes vulpes*) in Catalonia, northeastern Spain. Vet J.

[53] Demiaszkiewicz AW, Pyziel AM, Kuligowska I, Lachowicz J (2014). The first report of *Angiostrongylus vasorum* (Nematoda; Metastrongyloidea) in Poland, in red foxes (*Vulpes vulpes*). Acta Parasitol.

[54] Franssen F, Nijsse R, Mulder J, Cremers H, Dam C, Takumi K (2014). Increase in number of helminth species from Dutch red foxes over a 35-year period. Parasit Vectors.

[55] Lassnig H, Prosl H, Hinterdorfer F (1998). Parasites of the red fox (*Vulpes vulpes*) in Styria. Wien Tieraerztl Monatsschr.

[56] Saeed I, Maddox-Hyttel C, Monrad J, Kapel CM (2006). Helminths of red foxes (*Vulpes vulpes*) in Denmark. Vet Parasitol.

[57] Figueiredo A, Oliveira L, Madeira de Carvalho L, Fonseca C, Torres RT (2016). Parasite species of the endangered Iberian wolf (*Canis lupus signatus*) and a sympatric widespread carnivore. Int J Parasitol Parasites Wildl.

[58] Garrido-Castañé I, Ortuño A, Marco I, Castellà J (2015). Cardiopulmonary helminths in foxes from the Pyrenees. Acta Parasitol.

[59] Hodžić A, Alić A, Klebić I, Kadrić M, Brianti E, Duscher GG (2016). Red fox (*Vulpes vulpes*) as a potential reservoir host of cardiorespiratory parasites in Bosnia and Herzegovina. Vet Parasitol.

[60] Sréter T, Széll Z, Marucci G, Pozio E, Varga I (2003). Extraintestinal nematode infections of red foxes (*Vulpes vulpes*) in Hungary. Vet Parasitol.

[61] Lalošević V, Lalošević D, Čapo I, Simin V, Galfi A, Traversa D (2013). High infection rate of zoonotic *Eucoleus aerophilus* infection in foxes from Serbia. Parasite.

[62] Epe C, Coati N, Schnieder T (2004). Results of parasitological examinations of faecal samples from horses, ruminants, pigs, dogs, cats, hedgehogs and rabbits between 1998 and 2002. Dtsch Tieraerztl Wochenschr.

[63] Guardone L, Schnyder M, Macchioni F, Deplazes P, Magi M (2013). Serological detection of circulating *Angiostrongylus vasorum* antigen and specific antibodies in dogs from central and northern Italy. Vet Parasitol.

[64] Gillis-Germitsch N, Kapel CMO, Thamsborg SM, Deplazes P, Schnyder M (2017). Host-specific response to *Angiostrongylus vasorum* infection in red foxes (*Vulpes vulpes*): implications for parasite epidemiology. Parasitology.

[65] Patel Z, Gill AC, Fox MT, Hermosilla C, Backeljau T, Breugelmans K (2014). Molecular identification of novel intermediate host species of *Angiostrongylus vasorum* in greater London. Parasitol Res.

[66] Lange MK, Penagos-Tabares F, Muñoz-Caro T, Gärtner U, Mejer H, Schaper R (2017). Gastropod-derived haemocyte extracellular traps entrap metastrongyloid larval stages of *Angiostrongylus vasorum*, *Aelurostrongylus abstrusus* and *Troglostrongylus brevior*. Parasit Vectors.

[67] Magi M, Guardone L, Prati MC, Mignone W, Macchioni F (2015). Extraintestinal nematodes of the red fox *Vulpes vulpes* in north-west Italy. J Helminthol.

[68] Houpin E, McCarthy G, Ferrand M, De Waal T, O'Neill EJ, Zintl A (2016). Comparison of three methods for the detection of *Angiostrongylus vasorum* in the final host. Vet Parasitol.

[69] Bourque AC, Conboy G, Miller LM, Whitney H (2008). Pathological findings in dogs naturally infected with *Angiostrongylus vasorum* in Newfoundland and Labrador, Canada. J Vet Diagn Investig.

[70] Roche MM, Kelliher DJ (1968). *Angiostrongylus vasorum* infestation in a dog: a case report. Irish Vet J.

[71] Simpson VR, Neal C (1982). *Angiostrongylus vasorum* infection in snails and slugs. Vet Rec.

[72] Morgan ER, Jefferies R, Krajewski M, Ward P, Shaw SE (2009). Canine pulmonary angiostrongylosis: the influence of climate on parasite distribution. Parasitol Int.

[73] Jolly S, Poncelet L, Lempereur L, Caron Y, Bayrou C, Cassart D (2015). First report of a fatal autochthonous canine *Angiostrongylus vasorum* infection in Belgium. Parasitol Int.

[74] Pantchev N, Schnyder M, Vrhovec MG, Schaper R, Tsachev I (2015). Current surveys of the seroprevalence of *Borrelia burgdorferi*, *Ehrlichia canis*, *Anaplasma phagocytophilum*, *Leishmania infantum*, *Babesia canis*, *Angiostrongylus vasorum* and *Dirofilaria immitis* in dogs in Bulgaria. Parasitol Res.

[75] Taylor CS, Garcia Gato R, Learmount J, Aziz NA, Montgomery C, Rose H (2015). Increased prevalence and geographic spread of the cardiopulmonary nematode *Angiostrongylus vasorum* in fox populations in great Britain. Parasitology.

[76] DCK VD, Van de Sande AH, Nijsse ER, Eysker M, Ploeger HW (2009). Autochthonous *Angiostrongylus vasorum* infection in dogs in the Netherlands. Vet Parasitol.

[77] Alho AM, Schnyder M, Schaper R, Meireles J, Belo S, Deplazes P (2016). Seroprevalence of circulating *Angiostrongylus vasorum* antigen and parasite-specific antibodies in dogs from Portugal. Parasitol Res.

[78] Åblad B, Christensson D, Osterman Lind E, Ågren E, Mörner T (2003). *Angiostrongylus vasorum* etablerad i Sverige. Svensk Veterinartidning.

[79] Hurníková Z, Miterpáková M, Mandelík R (2013). First autochthonous case of canine *Angiostrongylus vasorum* in Slovakia. Parasitol Res.

[80] Colella V, Mutafchiev Y, Cavalera MA, Giannelli A, Lia RP, Dantas-Torres F (2016). Development of *Crenosoma vulpis* in the common garden snail *Cornu aspersum*: implications for epidemiological studies. Parasit Vectors.

[81] Tolnai Z, Széll Z, Sréter T (2015). Environmental determinants of the spatial distribution of *Angiostrongylus vasorum*, *Crenosoma vulpis* and *Eucoleus aerophilus* in Hungary. Vet Parasitol.

[82] Traversa D, Guglielmini C (2008). Feline aelurostrongylosis and canine angiostrongylosis: a challenging diagnosis for two emerging verminous pneumonia infections. Vet Parasitol.

