# Supplementary material for: Prevalence survey on lungworm (Angiostrongylus vasorum, Crenosoma vulpis, Eucoleus aerophilus) infections of wild red foxes (Vulpes vulpes) in central Germany
Source: Parasit Vectors. 2018 Feb 6;11:85. doi: 10.1186/s13071-018-2672-4 (PMC5801722; doi:10.1186/s13071-018-2672-4)
Supplement: Supplementary file 1 — Distribution of Angiostrongylus vasorum (Av), Crenosoma vulpis (Cv) and Eucoleus aerophilus (Ea) positive carcasses per county/city in the Federal State of Hesse (percentage and total numbers). (DOCX 18 kb) [file 13071_2018_2672_MOESM1_ESM.docx]

Additional file 1: Table S1. Distribution of *Angiostrongylus vasorum* (Av), *Crenosoma vulpis* (Cv) and *Eucoleus aerophilus* (Ea) positive carcasses per county/city in the Federal State of Hesse (percentage and total numbers)

| **County/City** | **Govern-mental district** | **Percentage Av positive in total (x/y)** | **Percentage Cv positive  in total (x/y)** | **Percentage Ea positive  in total (x/y)** | **Percentage Av+Cv positive (x/y)** | **Percentage Av+Ea positive (x/y)** | **Percentage Cv+Ea positive (x/y)** | **Percentage Av+Cv+Ea positive (x/y)** |
| --- | --- | --- | --- | --- | --- | --- | --- | --- |
| **Fulda** | **Kassel** | – (0/1) | – (0/1) | 100% (1/1) | – | – | – | – |
| **Kassel County** |  | 39.1% (9/23) | – (0/23) | 65.2% (15/23) | – | 34.8% (8/23) | – | – |
| **Schwalm-Eder-District** |  | – (0/1) | 100% (1/1) | 100% (1/1) | – | – | 100% (1/1) | – |
| **Waldeck-Frankenberg** |  | – (0/2) | 50% (1/2) | 100% (2/2) | – | – | 50% (1/2) | – |
| **Total** |  | 33.3% (9/27) | 7.4% (2/27) | 70.4% (19/27) | – | 29.6% (8/27) | 7.4% (2/27) | – |
| **Giessen County** | **Giessen** | 50% (1/2) | 50% (1/2) | 50% (1/2) | – | – | – | 50% (1/2) |
| **Lahn-Dill-District** |  | – (0/2) | 100% (2/2) | 100% (2/2) | – | – | 100% (2/2) | – |
| **Limburg-Weilburg** |  | 100% (2/2) | 100% (2/2) | 100% (2/2) | – | – | - | 100% (2/2) |
| **Marburg-Biedenkopf** |  | – (0/9) | 44.4% (4/9) | 100% (9/9) | – | – | 44.4% (4/9) | – |
| **Vogelsberg-District** |  | – (0/2) | 50% (1/2) | 100% (2/2) | – | – | 50% (1/2) | – |
| **Total** |  | 17.7% (3/17) | 58.8% (10/17) | 94.1% (16/17) | – | – | 41.2% (7/17) | 17.7% (3/17) |
| **Bergstraße** | **Darmstadt** | – (0/2) | – (0/2) | 100% (2/2) | – | – | – | – |
| **Darmstadt plus Darmstadt-Dieburg** |  | 27.3% (3/11) | 36.4% (4/11) | 81.8% (9/11) | – | 9.1% (1/11) | 27.3% (3/11) | 9.1% (1/11) |
| **Groß-Gerau** |  | – (0/4) | 75% (3/4) | 100% (4/4) | – | – | 75% (3/4) | – |
| **Main-Kinzig-District** |  | – (0/2) | – (0/2) | 100% (2/2) | – | – | – | – |
| **Main-Taunus-District** |  | 100% (1/1) | – (0/1) | 100% (1/1) | – | 100% (1/1) | – | – |
| **Wetterau-District** |  | – (0/8) | 37.5% (3/8) | 87.5% (7/8) | – | – | 37.5% (3/8) | – |
| **Wiesbaden** |  | – (0/3) | 66.7% (2/3) | 33.3% (1/3) | – | – | 33.3% (1/3) | – |
| **Total** |  | 12.9% (4/31) | 38.7% (12/31) | 83.9% (26/31) | – | 6.5% (2/31) | 32.3% (10/31) | 3.2% (1/31) |
| **Unknown** | **Unknown** | 7.1% (1/14) | 21.4% (3/14) | 21.4% (3/14) | 7.1% (1/14) | – | 14.3% (2/14) | – |
| **Total** | **Hesse** | **19.1% (17/89)** | **30.3% (27/89)** | **71.9% (64/89)** | **1.1% (1/89)** | **11.2% (10/89)** | **23.6% (21/89)** | **4.5% (4/89)** |

x: fox carcasses positive for a specific parasite, y: total number of foxes examined per county/city respectively governmental district
