# Supplementary material for: Prevalence survey on lungworm (Angiostrongylus vasorum, Crenosoma vulpis, Eucoleus aerophilus) infections of wild red foxes (Vulpes vulpes) in central Germany
Source: Parasit Vectors. 2018 Feb 6;11:85. doi: 10.1186/s13071-018-2672-4 (PMC5801722; doi:10.1186/s13071-018-2672-4)
Supplement: Supplementary file 2 — Distribution of Angiostrongylus vasorum (Av), Crenosoma vulpis (Cv) and Eucoleus aerophilus (Ea) positive carcasses per county/city in the Federal State of Rhineland-Palatinate (percentage and total numbers). (DOCX 17 kb) [file 13071_2018_2672_MOESM2_ESM.docx]

Additional file 2: Table S2. Distribution of *Angiostrongylus vasorum* (Av), *Crenosoma vulpis* (Cv) and *Eucoleus aerophilus* (Ea) positive carcasses per county/city in the Federal State of Rhineland-Palatinate (percentage and total numbers)

| **County/City** | **Geogra-phical region** | **Percentage Av positive  in total (x/y)** | **Percentage Cv positive  in total (x/y)** | **Percentage Ea positive  in total (x/y)** | **Percentage Av+Cv positive (x/y)** | **Percentage Av+Ea positive (x/y)** | **Percentage Cv+Ea positive (x/y)** | **Percentage Av+Cv+Ea positive (x/y)** |
| --- | --- | --- | --- | --- | --- | --- | --- | --- |
| **Ahrweiler** | **North** | – (0/3) | 33.3% (1/3) | 66.7% (2/3) | – | – | 33.3% (1/3) | – |
| **Altenkirchen (Westerwald)** |  | 40% (2/5) | 20% (1/5) | 40% (2/5) | – | – | – | 20% (1/5) |
| **Koblenz plus Mayen-Koblenz** |  | 30% (3/10) | 30% (3/10) | 80% (8/10) | – | – | 10% (1/10) | 20% (2/10) |
| **Neuwied** |  | – (0/1) | – (0/1) | 100% (1/1) | – | – | – | – |
| **Rhein-Lahn-District** |  | 14.3% (3/21) | 14.3% (3/21) | 81% (17/21) | – | 9.5% (2/21) | 9.5% (2/21) | 4.8% (1/21) |
| **Westerwald-District** |  | 10% (1/10) | 70% (7/10) | 100% (10/10) | – | – | 60% (6/10) | 10% (1/10) |
| **Total** |  | 18%  (9/50) | 30%  (15/50) | 80%  (40/50) | – | 4%  (2/50) | 20%  (10/50) | 10%  (5/50) |
| **Bad Kreuznach** | **Central** | – (0/1) | – (0/1) | 100% (1/1) | – | – | – | – |
| **Bernkastel-Wittlich** |  | 50% (1/2) | – (0/2) | 50% (1/2) | – | – | – | – |
| **Birkenfeld** |  | 8.3% (1/12) | 50% (6/12) | 83.3% (10/12) | – | – | 33.3% (4/12) | 8.3% (1/12) |
| **Eifel-District Bitburg-Prüm** |  | 10% (1/10) | 20% (2/10) | 60% (6/10) | – | – | 10% (1/10) | 10% (1/10) |
| **Cochem-Zell** |  | – (0/2) | 50% (1/2) | 100% (2/2) | – | – | 50% (1/2) | – |
| **Rhein-Hunsrück-District** |  | 50% (1/2) | 100% (2/2) | 100% (2/2) | – | – | 50% (1/2) | 50% (1/2) |
| **Trier plus  Trier-Saarburg** |  | 33.3% (1/3) | 33.3% (1/3) | 66.7% (2/3) | – | 33.3% (1/3) | 33.3% (1/3) | – |
| **Vulkaneifel** |  | – (0/5) | – (0/5) | 80% (4/5) | – | – | – | – |
| **Worms plus  Alzey-Worms** |  | – (0/2) | – (0/2) | 50% (1/2) | – | – | – | – |
| **Total** |  | 12.8%  (5/39) | 30.8%  (12/39) | 74.4%  (29/39) | – | 2.6%  (1/39) | 20.5%  (8/39) | 7.7%  (3/39) |
| **Bad Dürkheim** | **South** | 100% (1/1) | 100% (1/1) | 100% (1/1) | – | – | – | 100% (1/1) |
| **Donnersberg-District** |  | 85.7% (6/7) | 14.3% (1/7) | 71.4% (5/7) | 14.3% (1/7) | 57.1% (4/7) | – | – |
| **Germersheim** |  | – (0/1) | – (0/1) | 100% (1/1) | – | – | – | – |
| **Kaiserslautern plus Kaiserslautern County** |  | 100% (2/2) | – (0/2) | 50% (1/2) | – | 50% (1/2) | – | – |
| **Kusel** |  | 14.3% (1/7) | – (0/7) | 57.1% (4/7) | – | – | – | – |
| **Rhein-Pfalz-District** |  | 60% (3/5) | 20% (1/5) | 60% (3/5) | – | 40% (2/5) | – | 20% (1/5) |
| **Südliche Weinstraße** |  | 50% (1/2) | 50% (1/2) | 100% (2/2) | – | 50% (1/2) | 50% (1/2) | – |
| **Südwestpfalz** |  | 71.4% (5/7) | – (0/7) | 71.4% (5/7) | – | 42.9% (3/7) | – | – |
| **Total** |  | 59.4%  (19/32) | 12.5%  (4/32) | 68.8%  (22/32) | 3.1%  (1/32) | 34.4%  (11/32) | 3.1%  (1/32) | 6.3%  (2/32) |
| **Total** | **Rhinel.-Palat.** | **27.3%  (33/121)** | **25.6%  (31/121)** | **75.2%  (91/121)** | **0.8%  (1/121)** | **11.6%  (14/121)** | **15.7%  (19/121)** | **8.3%  (10/121)** |

x: fox carcasses positive for a specific parasite, y: total number of foxes examined per county/city respectively geographical region
