# Supplementary material for: Prevalence survey on lungworm (Angiostrongylus vasorum, Crenosoma vulpis, Eucoleus aerophilus) infections of wild red foxes (Vulpes vulpes) in central Germany
Source: Parasit Vectors. 2018 Feb 6;11:85. doi: 10.1186/s13071-018-2672-4 (PMC5801722; doi:10.1186/s13071-018-2672-4)
Supplement: Supplementary file 3 — Distribution of Angiostrongylus vasorum (Av), Crenosoma vulpis (Cv) and Eucoleus aerophilus (Ea) positive carcasses per county/city in the Federal State of Thuringia (percentage and total numbers). (DOCX 17 kb) [file 13071_2018_2672_MOESM3_ESM.docx]

Additional file 3: Table S3. Distribution of *Angiostrongylus vasorum* (Av), *Crenosoma vulpis* (Cv) and *Eucoleus aerophilus* (Ea) positive carcasses per county/city in the Federal State of Thuringia (percentage and total numbers)

| **County/City** | **Geogra-phical region** | **Percentage Av positive  in total (x/y)** | **Percentage Cv positive  in total (x/y)** | **Percentage Ea positive  in total (x/y)** | **Percentage Av+Cv positive (x/y)** | **Percentage Av+Ea positive (x/y)** | **Percentage Cv+Ea positive (x/y)** | **Percentage Av+Cv+Ea positive (x/y)** |
| --- | --- | --- | --- | --- | --- | --- | --- | --- |
| **Eichsfeld** | **North** | 16.7% (2/12) | 8.3% (1/12) | 75% (9/12) | – | 16.7% (2/12) | 8.3% (1/12) | – |
| **Kyffhäuser-District** |  | 13% (3/23) | 30.4% (7/23) | 78.3% (18/23) | – | 8.7% (2/23) | 26.1% (6/23) | – |
| **Nordhausen** |  | 16.7% (2/12) | 16.7% (2/12) | 75% (9/12) | – | – | 16.7% (2/12) | – |
| **Unstrut-Hainich-District** |  | 13.5% (7/52) | 28.9% (15/52) | 48.1% (25/52) | 1.9% (1/52) | 3.8% (2/52) | 19.2% (10/52) | 1.9% (1/52) |
| **Total** |  | 14.1%  (14/99) | 25.3%  (25/99) | 61.6%  (61/99) | 1%  (1/99) | 6.1%  (6/99) | 19.2%  (19/99) | 1%  (1/99) |
| **Eisenach plus Wartburg-District** | **West** | – (0/16) | 12.5% (2/16) | 81.3% (13/16) | – | – | 12.5% (2/16) | – |
| **Hildburghausen** |  | 8.3% (2/24) | 29.2% (7/24) | 75% (18/24) | – | 4.2% (1/24) | 25% (6/24) | 4.2% (1/24) |
| **Schmalkalden-Meiningen** |  | – (0/44) | 36.4% (16/44) | 77.3% (34/44) | – | – | 31.8% (14/44) | – |
| **Sonneberg** |  | 7.7% (1/13) | 38.5% (5/13) | 92.3% (12/13) | – | 7.7% (1/13) | 38.5% (5/13) | – |
| **Suhl** |  | – (0/3) | 33.3% (1/3) | 33.3% (1/3) | – | – | 33.3% (1/3) | – |
| **Total** |  | 3%  (3/100) | 31% (31/100) | 78%  (78/100) | – | 2%  (2/100) | 28%  (28/100) | 1%  (1/100) |
| **Altenburger Land** | **East** | 11.1% (1/9) | 44.4% (4/9) | 77.8% (7/9) | – | – | 33.3% (3/9) | – |
| **Gera plus Greiz** |  | 11.1% (2/18) | 50% (9/18) | 55.6% (10/18) | – | – | 16.7% (3/18) | 11.1% (2/18) |
| **Jena plus Saale-Holzland-District** |  | – (0/30) | 53.3% (16/30) | 60% (18/30) | – | – | 36.7% (11/30) | – |
| **Saale-Orla-District** |  | 12.5% (2/16) | 50% (8/16) | 68.8% (11/16) | – | 6.3% (1/16) | 37.5% (6/16) | – |
| **Total** |  | 6.9%  (5/73) | 50.7%  (37/73) | 63%  (46/73) | – | 1.4%  (1/73) | 31.5%  (23/73) | 2.7%  (2/73) |
| **Erfurt** | **Central** | – (0/6) | 33.3% (2/6) | 33.3% (2/6) | – | – | 16.7% (1/6) | – |
| **Gotha** |  | 20% (4/20) | 40% (8/20) | 65% (13/20) | – | 10% (2/20) | 30% (6/20) | 10% (2/20) |
| **Ilm-District** |  | 16.7% (3/18) | 55.6% (10/18) | 72.2% (13/18) | – | 11.1% (2/18) | 50% (9/18) | 5.6% (1/18) |
| **Saalfeld-Rudolstadt** |  | 3.7% (1/27) | 48.2% (13/27) | 63% (17/27) | – | – | 33.3% (9/27) | 3.7% (1/27) |
| **Sömmerda** |  | – (0/11) | – (0/11) | 45.5% (5/11) | – | – | – | – |
| **Weimarer Land** |  | – (0/1) | – (0/1) | 100% (1/1) | – | – | – | – |
| **Total** |  | 9.6%  (8/83) | 39.8%  (33/83) | 61.5%  (51/83) | – | 4.8%  (4/83) | 30.1%  (25/83) | 4.8%  (4/83) |
| **Unknown** | **Unknown** | – (0/4) | – (0/4) | 100% (4/4) | – | – | – | – |
| **Total** | **Thuringia** | **8.4%  (30/359)** | **35.1%  (126/359)** | **66.9%  (240/359)** | **0.3%  (1/359)** | **3.6% (13/359)** | **26.5% (95/359)** | **2.2%  (8/359)** |

x: fox carcasses positive for a specific parasite, y: total number of foxes examined per county/city respectively geographical region
